# Data resource profile: Transforming Outcomes through Research in Cancer Healthcare in Victoria (TORCH-VIC)

**DOI:** 10.23889/ijpds.v8i6.3141

**Published:** 2026-05-21

**Authors:** Fanny Franchini, Karen Trapani, Jennifer Soon, Piers Gillett, Koen Degeling, Benjamin Daniels, Sophy Athan, Sallie-Anne Pearson, Maarten IJzerman

**Affiliations:** 1 Cancer Health Services Research, Collaborative Centre for Genomic Cancer Medicine, Faculty of Medicine, Dentistry and Health Sciences, The University of Melbourne, Melbourne, Australia; 2 Cancer Health Services Research, Centre for Health Policy, Melbourne School of Population and Global Health, Faculty of Medicine, Dentistry and Health Sciences, The University of Melbourne, Melbourne, Australia; 3 Sir Peter MacCallum Department of Oncology, Faculty of Medicine, Dentistry and Health Sciences, The University of Melbourne, Melbourne, Australia; 4 Medicines Intelligence Research Program, School of Population Health, UNSW Sydney, Sydney New South Wales, Australia; 5 Victorian Comprehensive Cancer Centre Alliance, Melbourne, Australia; 6 Erasmus School of Health Policy and Management, Rotterdam, the Netherlands; † These authors contributed equally.

**Keywords:** data linkage, cancer care, drug prescriptions, health equity, epidemiology, geography, Australia, health technology assessment, public health, administrative data research

## Abstract

**Introduction:**

The Transforming Outcomes through Research in Cancer Healthcare in Victoria (TORCH-VIC) is a comprehensive population-based cohort that links cancer diagnoses recorded in the Victorian Cancer Registry with administrative health data to capture the complete cancer journey from pre-diagnosis through long-term outcomes. Established in 2022, TORCH-VIC provides real-world evidence on healthcare utilisation, patterns of care, costs, and outcomes across the cancer continuum in Victoria, Australia.

**Methods:**

TORCH-VIC comprises adults aged 18 years and over diagnosed with colorectal, lung, melanoma, prostate, breast cancer, and lymphoid leukaemia in Victoria, Australia, between January 2010 and December 2021, identified through the Victorian Cancer Registry. Cross-jurisdictional linkage of 11 data collections was performed by the Centre for Victorian Data Linkage for state datasets (hospital admissions, emergency presentations, radiotherapy, outpatient services, elective surgery, costs, and deaths) and the Australian Institute of Health and Welfare Data Linkage Unit for national datasets (Pharmaceutical Benefits Scheme (PBS), Medicare Benefits Schedule (MBS), National Death Index). Annual refresh ensures ongoing data currency.

**Results:**

The dataset contains 222,332 unique individuals with 237,089 cancer diagnoses, stored securely in the Secure Unified Research Environment (SURE). Linkage rates exceed 98% for PBS and MBS data, with comprehensive inpatient hospital coverage (98.7%). The dataset captures over 250 million healthcare interactions across four phases: pre-diagnosis pathways, diagnosis, interventions, and outcomes. Data domains include socio-demographics, mortality, comorbidities, healthcare services, medicines, and costs. Mortality is comprehensively captured through linked state and national death registries (35.0% of cohort deceased). Loss to follow-up is minimal (1%), occurring primarily among individuals who emigrate or are ineligible for Medicare, with national datasets ensuring continued follow-up for those who relocate interstate within Australia.

**Conclusion:**

TORCH-VIC enables research on cancer epidemiology, health service delivery, disparities in access, supportive care utilisation, survivorship, and healthcare costs. Applications span health services research, policy evaluation, and outcomes assessment. With ethics approvals secured, 2026 enhancements will add screening registers, immunisation data, and additional cancer types. Researchers interested in collaboration should contact the corresponding author to discuss projects within the study scope and ethics requirements.

## Key features

- The Transforming Outcomes through Research in Cancer Healthcare in Victoria (TORCH-VIC) cohort was established to address gaps in comprehensive real-world evidence on the cancer care continuum in Australia, with a unique focus on capturing patterns of care, costs, and outcomes.
- TORCH-VIC includes 222,332 adults with a cancer diagnosis for colorectal, lung, melanoma, prostate, breast cancer, and lymphoid leukaemia between 2010-2021. Age at diagnosis ranges from 18-94 years (median 71). Females account for 45.2% of the current cohort, and 66.9% of individuals were born in Australia.
- The data is refreshed annually with comprehensive data linkage across state and national administrative datasets, minimising loss to follow-up.
- This linkage is the first Victorian linkage of population-based cancer diagnoses to all prescription medicines and medical services under Medicare. Main data categories include cancer diagnoses, hospital admitted, non-admitted and emergency presentations, prescription medicines, medical services, radiotherapy treatments, elective surgery information, death records, and healthcare costs.
- TORCH-VIC welcomes researchers interested in collaborative project proposals. Interested research collaborators can contact the corresponding author for more detail.

## Background

Large-scale linked datasets combining cancer registry information with administrative health data have become essential infrastructure for cancer health services research and policy. These resources enable comprehensive examination of the cancer care continuum, from diagnostic pathways and treatment patterns through to survivorship, costs, and long-term outcomes. They can also help generate evidence to inform clinical practice guidelines, health technology assessment, health economics, and health system planning.

Internationally, linked cancer datasets combining registry information with administrative health data have been established across diverse healthcare systems. Countries with unique personal identifiers and centralised data systems, such as Denmark [1] and Taiwan [2, 3], achieve near-universal linkage across cancer registrations, hospital care, and prescription data. In the United Kingdom, national cancer registry linkages to Hospital Episode Statistics and the Systemic Anti-Cancer Therapy (SACT) dataset provide whole-of-population coverage within a single-payer system [4]. SEER-Medicare in the United States of America, established in 1991, combines cancer registry data with Medicare claims but is restricted to adults aged 65 years and older in fee-for-service Medicare [5, 6]. Countries with federated healthcare systems, including Canada and Australia, face additional challenges in achieving cross-jurisdictional data harmonisation, with provincial and state-level registries maintaining varying levels of treatment data linkage [7].

Australia has a universal healthcare coverage system providing publicly funded access to prescription medicines through the national Pharmaceutical Benefits Scheme (PBS), medical services through the Medicare Benefits Schedule (MBS), and hospital care, for which extensive administrative data collections are maintained to provide reimbursement for these services. Despite recent advancements in Australian health data linkage capabilities, such as the National Health Data Hub [8] or Person Level Integrated Data Asset [9], there remains no established infrastructure for large-scale linkage of cancer registry data with treatment and outcome information at a national level that includes all Australian jurisdictions. As a result, there are significant gaps in understanding diagnostic pathways, quantifying the number of treatments provided to patients, characterising patterns of care beyond first-line settings, examining survivorship and long-term outcomes, and estimating costs of cancer care for patients and payers.

The Transforming Outcomes through Research in Cancer Healthcare in Victoria (TORCH-VIC) cohort was established in 2022 to address this gap within Victoria, Australia’s second most populous state. Victoria is located in southeastern Australia, with an estimated population of 7 million representing approximately 25% of the national population [10]. The state capital, Melbourne, is Australia’s second-largest city (5.3 million people), and home to approximately 75% of Victoria’s population, with the remainder in regional and rural areas [10]. Victoria has a large population base, with a mix of urban and regional communities, socioeconomic, and cultural diversity, which makes it well-suited for cancer health services research, including for examining equity in access to care and outcomes.

The primary aims of the TORCH-VIC cohort are 1) to determine the type and frequency of diagnostic tests and treatments used in contemporary Victorian clinical practice, 2) to estimate the costs of cancer care, 3) to identify disparities in access and provision of care, and 4) to study adverse events and multi morbidity in the cancer setting.

The foundation of TORCH-VIC is the Victorian Cancer Registry (VCR), a population-based registry responsible for accurate and timely reporting of cancer incidence, mortality and survival in Victoria [11]. Under the Victorian legislation Improving Cancer Outcomes Act 2014 and its related regulations, all Victorian hospitals, pathology, and radiotherapy services are required to report to the VCR the details of all persons with a cancer diagnosis [12]. This mandatory reporting ensures whole-of-population coverage of all people diagnosed with cancer in Victoria. The VCR is complemented by comprehensive state-based administrative datasets including hospital admissions, emergency presentations, radiotherapy treatments, elective surgery, and death records, alongside national datasets capturing prescription medicines and medical services. TORCH-VIC connects these data sources through cross-jurisdictional linkage, bridging state and national systems to maximise the public benefit derived from these comprehensive data collections.

TORCH-VIC was initially developed to support the Predicting the population health economic impact of current and new cancer treatments (PRIMCAT) research program [13], which addressed national Health Technology Assessment (HTA) policy challenges and data gaps. PRIMCAT aimed to support Australian HTA agencies with validated epidemiological data and models [14] to support subsidy decision making and streamline access to new cancer treatments for Australians affected by cancer [15]. Consumer representatives participated in the initial research prioritisation and contributed to TORCH-VIC data development and governance, ensuring that the research agenda reflects their perspectives and community benefit. The seed funding for the TORCH-VIC cohort (also known as the PRIMCAT dataset) came from the PRIMCAT research program, funded by the Medical Research Future Fund (grant number 2020/MRF1199701).

## Methods

TORCH-VIC is a population-based linked data resource covering all adults (aged ≥ 18 years) diagnosed with colorectal, lung, melanoma, prostate, breast cancer, and lymphoid leukaemia in Victoria, Australia, between 1 January 2010 and 31 December 2021. Specifically, these include the five most commonly diagnosed cancers in Australia, which collectively account for over half of all cancer diagnoses, and lymphoid leukaemia to provide representation of haematological malignancies. These cancer types encompass diverse treatment modalities and have experienced substantial therapeutic advances, making them priorities for evidence generation from routinely collected health data. Data collections for linkage were selected based on availability within the Australian health data infrastructure, coverage of key domains across the cancer care continuum, and alignment with the study aims. Cohort members were identified through the Victorian Cancer Registry (VCR). The cohort comprises individual-level administrative and registry data from three national and eight Victorian (state-based) data collections. All data sources linked in TORCH-VIC are described in Table 1, which also presents a data coverage timeline. Cohort identification is based on VCR cancer diagnoses from January 2010 to December 2021, however, the temporal coverage of linked data collections extends beyond this period. PBS and MBS data are available from January 2008, enabling analysis of pre-diagnosis healthcare utilisation, while most other collections extend to mid-2023, providing continued follow-up of outcomes.

**Table 1:** Description and data coverage timelines of the linked data collections in TORCH-VIC

**Data collection**	**Acronym**	**Time period**	**Key information (including coding systems)**
Victorian Cancer Registry	VCR	Jan 2010 – Dec 2021	Population-based cancer registry providing comprehensive, accurate and timely information for cancer control. All Victorian hospitals, pathology services and prescribed registers, are required to notify details of patients admitted and treated for cancers reportable under the Cancer Act, 1958, as amended [11]. Contains demographic data and clinical information on the cancer type, including date of diagnosis, derived stage at diagnosis, and specific information such as ER and HER2 status for breast cancer, Gleason score for prostate cancer, and melanoma level and thickness [16].
Victorian Admitted Episodes Dataset	VAED	Jan 2010 – May 2023	Includes admitted episodes of care in Victorian public and private hospitals, rehabilitation centres, extended care facilities and day procedure centres [17]. Contains demographic, clinical and administrative details for admitted episode of care, with diagnoses coded with information from the International Classification of Diseases, Tenth Revision, Australian Modification (ICD-10-AM) and procedures coded with the Australian Classification of Health Interventions (ACHI).
Victorian Emergency Minimum Dataset	VEMD	Jan 2010 – May 2023	Comprises demographic, administrative, and clinical data detailing presentations at Victorian public hospitals with designated emergency departments. The VEMD does not capture emergency department presentations at private hospitals [18]. Contains information on patient demographics including residential location, diagnosis (ICD-10-AM) and procedures (ACHI), arrival and departure details.
Victorian Integrated Non-Admitted Health	VINAH	Jan 2010 – May 2023	Comprises data about non-admitted services including the Hospital Admission Risk Program (HARP), Specialist Clinics (Outpatients), Palliative Care (PC), Post-Acute Care (PAC), Subacute Ambulatory Care Services (SACS), Victorian HIV Service (VHS) and the Victorian Respiratory Support Service (VRSS) [19]. Contains information related to patient demographics, referral details, medical condition, service provided and associated dates.
Victorian Radiotherapy Minimum Data Set	VRMDS	Jan 2010 – May 2023	Comprises demographic, administrative, and clinical data for admitted and non-admitted patients treated in Victorian Radiotherapy facilities in the public and private sector [20]. Contains information on patient level demographics including geographic location, treatment details, type and site of radiotherapy.
Victorian Death Index	VDI	Jan 2010 – May 2023	VID data is obtained from the Victorian Registry of Births Deaths and Marriages [21], under a Memorandum of Understanding. Contains information on the date of death, age at death, cause of death, sex of deceased, number of siblings, marital status at death, number and age of children, last residence of deceased.
Elective Surgery Information System	ESIS	Jan 2010 – May 2023	Patient level collection of elective surgery waiting list data from approved Victorian public healthcare services. Elective surgery is planned surgery that can be booked in advance because of a specialist clinical assessment resulting in placement on an elective surgery waiting list [22]. Contains information on demographics including residential location, diagnosis (ICD-10-AM) and procedures (ACHI), arrival and departure details.
Victorian Cost Data Collection	VCDC	Jan 2010 – Dec 2022	Reflects the cost and mix of resources used to deliver patient care. Victorian public hospitals are required to report costs for all operational funded activity and are expected to maintain patient costing systems that monitor service provision to patients and allow for the accurate determination of patient level costs [23]. Key costing data is available by Community Care Unit, Emergency, Intensive Care Unit, Imaging, Medical, Surgery, Medical Non-Surgery, Nursing, Pathology, Pharmacy, Theatre, Allied and Total cost.
Pharmaceutical Benefits Scheme data collection	PBS	Jan 2008 – June 2023	Contains information on all prescription medicines dispensed anywhere in Australia that qualify for a benefit under the National Health Act 1953 and for which a claim has been processed [24]. Contains information on the medicine dispensed, **including cancer therapeutics**, its strength, date of prescription and supply, and associated Anatomical Therapeutic Chemical (ATC) five-level hierarchical classification scheme. It also includes data related to location postcode, fees, prescribers, and dispensing pharmacies.
Medicare Benefits Schedule data collection	MBS	Jan 2008 – June 2023	Contains information on all medical services performed anywhere in Australia services that qualify for a benefit under the Health Insurance Act 1973 and for which a claim has been processed [25]. Contains information on the type of service provided, including consultations with general practionners and specialists, pathology tests, diagnostics and therapeutic procedures. It also includes data related to location postcode, fees, and service providers.
National Death Index	NDI	Jan 2010 – Dec 2022	Records all deaths occurring in Australia since 1980, supplied by the Registry of Births, Deaths and Marriages from each State or Territory [26]. Contains the primary underlying cause of death and additional causes of death as recorded on the death certificate. Causes of death are ICD-10 coded by the Australian Bureau of Statistics.
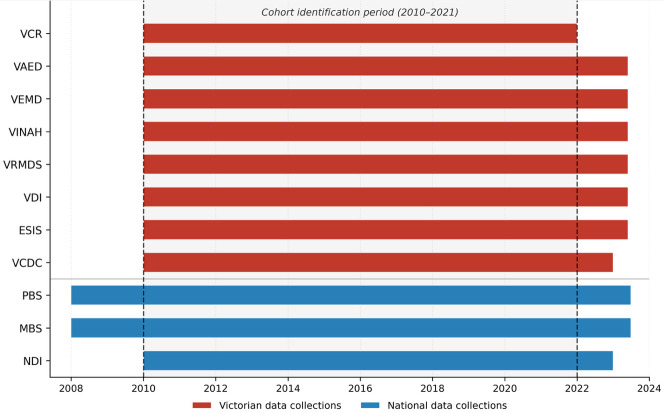			

### Data linkage and quality

TORCH-VIC undergoes a cross-jurisdictional linkage with an annual refresh process. Linkage is executed by the Centre for Victorian Data Linkage (CVDL) for Victorian-based administrative datasets [27], and by the Australian Institute of Health and Welfare (AIHW) Data Linkage Unit for National datasets [28], including prescription and medical services. Both CVDL and AIHW use standard operating procedures in line with best practices in data linkage, including the separation principle.

Cross-jurisdictional linkage between Victorian and Commonwealth datasets was undertaken using probabilistic record linkage with clerical review of uncertain matches. Of the 227,425 unique individuals provided by CVDL, 225,614 (99%) were successfully linked to national datasets (AIHW linkage report). Following linkage, the research team undertook additional data cleaning and consolidation across all datasets, including resolution of duplicate identifiers. This resulted in a final cohort of 222,332 unique persons. Only these individuals are included in all subsequent analyses. Deceased individuals remain in the cohort with mortality status and cause of death captured. For individuals with multiple cancer diagnoses, each cancer type can be analysed independently as the VCR assigns unique identifiers for cancer diagnosis.

Data quality in TORCH-VIC is ensured through several mechanisms. Loss to follow-up is minimal, occurring primarily among individuals who emigrate or are ineligible for Medicare, representing approximately 1% of the TORCH-VIC population. When individuals move interstate, their prescription medicines and medical services are recorded through the national coverage of PBS and MBS data, ensuring ongoing follow-up regardless of location within Australia. Cross-validation is performed for mortality data through multiple sources (VDI, NDI, and hospital records) and for sociodemographic information harmonised across all linked datasets.

### Data framework and coverage

TORCH-VIC provides comprehensive coverage of healthcare interactions across the entire cancer care continuum. The annual data refresh process allows for the longitudinal examination of cancer care trajectories across four distinct phases:

1. Pre-diagnosis phase: pathways to diagnosis, access to cancer screening and diagnostics, comorbidity status, and health services and medicines dispensed prior to diagnosis;
2. Diagnosis phase: baseline assessment of socio-demographics, clinical characteristics, and health services utilisation;
3. Treatment and intervention phase: analysis of timing and patterns of cancer treatment following diagnosis, including potential deviations from standards of care;
4. Outcomes phase: evaluation of disease progression, treatment changes, adverse events, comorbidities, health services utilisation, costs, and survival rates.

To support comprehensive analyses across these four phases, TORCH-VIC integrates multiple data domains, each providing essential components of the cancer care continuum:

- Sociodemographic. We harmonised and cross-validated sociodemographic and geographic information obtained from all data sources. This includes date of birth (month/year), sex, country and region of birth, language spoken at home, requirement for interpreter at points of care, marital status, socioeconomic status (SEIFA [29]), residence postcode, including related mapping to remoteness [30] and Monash Modified Model [31], and health services campuses and postcodes. Travel distance and time estimates to seek cancer care are analysed to identify potential barriers to access and burden of travel, especially for individuals living in remote and regional areas. All data are consolidated both at diagnosis and updated at various points across the continuum of care.
- Mortality and survival. We integrate data from the Victorian Death Index (VDI) and the National Death Index (NDI) to determine overall survival and detailed causes of mortality. This dual-source approach ensures a cross-validation of outcomes and comprehensive mortality data. Additionally, mortality is tracked through hospital admitted episodes of care (VAED), emergency episodes (VEMD), and non-admitted episodes (VINAH), as these data sources are more up to date than VDI and NDI.
- Comorbidity. We comprehensively track comorbidity through hospital data ICD-10AM codes (VAED, VEMD), from which Charlson Comorbidity Index scores are calculated, and ATC codes in prescription records (PBS) as proxy to provide an overview of each individual’s health status before and after a cancer diagnosis, and to assess how comorbidities may affect cancer treatment and outcomes.
- Cancer-related treatment. Linked PBS and MBS data, obtains historical information on publicly subsidised medicine exposures and outpatient treatments. As of April 2025, there were 154 unique PBS-subsidised [32] antineoplastic agents and endocrine therapies used in the cancer setting (ATC levels L01 and L02). These are complemented by granular and specialised data for radiotherapy treatment within VRMDS and by hospital data for surgical treatments and medical procedures through VAED, ESIS, and VINAH. While explicit treatment completion status is not a recorded variable in administrative datasets, treatment discontinuation or non-completion can be inferred from gaps in PBS dispensing records relative to expected treatment durations.
- Health and wellbeing support services. We capture utilisation of mental health services through MBS items, including psychology consultations (Better Access initiatives) and psychiatry services. Allied health services subsidised through Medicare are captured where applicable (for example chronic disease management plans). VINAH data provides additional coverage through its contact purpose classifications, including therapy/clinical interventions, education/self-management, social support, and case management services delivered through specialist clinics, HARP, and other ambulatory care programs. This enables examination of multidisciplinary supportive care utilisation, which research shows correlates with improved cancer outcomes and quality of life.
- Individual and healthcare costs. TORCH-VIC captures healthcare costs from multiple perspectives: person out-of-pocket expenses (co-payments PBS and MBS), government reimbursements for medications and services (PBS and MBS), and hospital service delivery costs (VCDC). Additionally, travel costs to access cancer care are estimated using geographic and distance data, enabling analysis of the financial burden of travel, particularly for those in regional and remote areas.

## Results

### Sociodemographic characteristics

Table 2 summarises the demographic characteristics of TORCH-VIC compared to the Victorian adult population (2021 census). The cohort includes 222,332 unique people with cancer diagnoses between January 2010 and December 2021. Males comprised 54.8% of TORCH-VIC, contrasting with the female majority in the general population. The median age was 71 years (versus 45 in Victoria), with 74% aged 65 years and older, reflecting the age distribution of cancer incidence. Geographic distribution showed lower metropolitan residence (70.0% vs 77.1%) and higher representation in inner regional areas (24.1% vs 18.7%). TORCH-VIC population had greater socioeconomic disadvantage, with 20.9% in the most disadvantaged SEIFA quintile compared to 14.7% state-wide. Australian-born individuals comprised 66.9% of the cohort, which was higher than the Victorian general population (59.3%).

**Table 2:** Demographic characteristics of TORCH-VIC cohort at time of first cancer diagnosis compared to Victoria, Australia

Characteristics	**^a^** **Victorian adult population, n (%)**	**^b^** ** VIC cohort, n (%)**
**Overall population**	5 114 719	222 332
Deaths	42 513 (0.83%)	77 856 (35.0%)
**Sex**		
Female	2 627 263 (51.4%)	100 423 (45.2%)
Male	2 487 456 (48.6%)	121 909 (54.8%)
**Age (median)**	45	71
**Age categories**		
18-24	554 218 (10.8%)	104 (0.04%)
25-34	975 505 (19.1%)	1 313 (0.6%)
35-44	918 737 (18.0%)	5 179 (2.3%)
45-54	826 890 (16.2%)	15 654 (7.0%)
55-64	746 549 (14.6%)	35 850 (16.1%)
65-74	605 554 (11.8%)	61 961 (27.8%)
75-84	344 801 (6.7%)	64 744 (29.1%)
85+	142 465 (2.8%)	37 990 (17.1%)
**Country of birth – Australia**	3 032 836 (59.3%)	149 129 (66.9%)
**Remoteness**		
Metropolitan	3 943 215 (77.1%)	155 775 (70.0%)
Inner regional	954 635 (18.7%)	53 668 (24.1%)
Outer regional	206 370 (4%)	13 117 (5.9%)
Remote	2 739 (0.1%)	228 (0.1%)
Missing/Not applicable	7 773 (0.2%)	7 (0.002%)
**Socio-economic disadvantage index (SEIFA)**		
1 (most disadvantaged)	753 694 (14.7%)	46 540 (20.9%)
2	926 448 (18.1%)	44 251 (19.9%)
3	1 072 956 (21.0%)	43 353 (19.5%)
4	1 175 381 (23.0%)	42 830 (19.2%)
5 (least disadvantaged)	1 178 354 (23.0%)	44 367 (19.9%)
Missing/Not applicable	7 886 (0.2%)	1 454 (0.6%)

^a^Overall Victorian adult population (18 years and over) based on the 2021Australian Census of Population and Housing, the most recent census available at the time of analysis. Australian census data are collected at five-year intervals; the comparison is indicative of population-level differences rather than a precise temporal match to the full TORCH-VIC cohort period (2010-2021).

^b^ TORCH-VIC adult population characteristics (18 years and over) were mapped to the relevant census year (2011, 2016, or 2021) based on the year of cancer diagnosis.

Table 3 shows both breadth of coverage (percentage of cohort with records) and depth (volume of records). Over the entire follow-up period, nearly all individuals (98.5%) had PBS medicine dispensing records, with cardiovascular medicines being the most frequently dispensed (36.6% of all dispensing), followed by nervous system (16.6%) and alimentary tract medicines (14.9%). Similarly, 98.3% accessed MBS services, predominantly for pathology (37.4% of all services) and GP or specialist consultations (31.3%). Hospital utilisation was high, with 98.7% having at least one inpatient admissions (VAED), 77.4% presenting to emergency departments (VEMD), and 76.4% who had an outpatient episode of care (VINAH). Radiotherapy had the lowest coverage, with 37.4% people who had least one radiotherapy-related healthcare activity.

**Table 3:** Prescription medicine, health service use, and major events in administrative data collected in TORCH-VIC population

Data collection	**Number of people with ≥ 1 health event (%)**	**Top 5 most common (% of total number of population with ≥ 1 record/event)**	**Total records**	**Top 5 most common by volume (% of all records)**
Total population TORCH-VIC	222 332 (100%)			
PBS medicine dispensing	219 010 (98.5%)	ATC main group anti-infectives (97.4%), nervous system (93.8%), alimentary (86.9%), cardiovascular (80.2%), musculoskeletal (72.7%)	88 886 500	ATC main group cardiovascular (36.6%), nervous system (16.6%), alimentary (14.9%), anti-infectives (5.6%), antineoplastics (5.1%)
MBS health service claims	218 661 (98.3%)	MBS category Pathology (99.7%), GP or specialist consultation (99.6%), diagnostic imaging (98.4%), therapeutic procedures (94.5%), miscellaneous (93.4%)	135 334 856	MBS category Pathology (37.4%), GP or specialist consultation (31.3%), miscellaneous (15.4%), therapeutic procedures (8.4%), diagnostic imaging (5.6%)
Hospital inpatient stay (VAED)	219 532 (98.7%)	Primary diagnosis neoplasms (91.3%), factors influencing health and contact with health services (58.7%), symptoms signs and abnormal findings (51.4%), digestive system (48.7%), circulatory system (31.9%)	Overall: 25 106 431 Unique admission: 3 864 226	Primary diagnosis Factors influencing health and contact with health services (44.1%), neoplasms (16.3%), digestive system (6.2%), symptoms signs and abnormal findings (6.0%), circulatory system (4.2%)
Emergency department attendance (VEMD)	172 107 (77.4%)	Triage category urgent (74.6%), semi-urgent (66.2%), emergency (43.9%), non-urgent (24.3%), resuscitation (3%)	Overall: 1 518 814 Unique admission: 870 182	Triage category urgent (42.6%), semi-urgent (32.9%), emergency (16.4%), non-urgent (7.4%), resuscitation (0.6%)
Hospital non-admitted episode (VINAH)	169 868 (76.4%)	Contact program stream oncology (56.2%), pre-admission (39.3%), general surgery (32.2%), allied health (31.9%), post-acute care (23.4%)	15 114 803	Contact program stream HARP complex care (14.4%), oncology (13.7%), post-acute care (10.6%), rehabilitation (8.5%), community palliative care (7.4%)
Radiotherapy treatment (VRMDS)	83 159 (37.4%)	Radiotherapy target (first site) breast (10.1%), chest and lung (7.7%), prostate (7.2%), spine (5.7%), brain (5.0%)	126 157	Radiotherapy target (first site) breast (8.6%), chest and lung (7.2%), brain (5.1%), spine (5.1%), prostate (5.0%)

PBS: Pharmaceutical Benefits Scheme; MBS: Medicare Benefits Schedule; VAED: Victorian Admitted Episodes Dataset; VEMD: Victorian Emergency Minimum Dataset; VINAH: Victorian Integrated Non-Admitted Health; VRMDS: Victorian Radiotherapy Minimum Dataset.

### Cancer-specific characteristics

Table 4 provides an overview of TORCH-VIC composition by cancer type, forming the foundation for data collection and analysis. The population comprises 222,332 people and 237,089 cancers between January 2010 and December 2021. Specifically, there were 52,634 people diagnosed with breast cancer, 62,120 with prostate cancer, 47,145 with colorectal cancer, 36,207 with lung cancer, 31,896 with melanoma, and 7,087 with lymphoid leukaemia. Some individuals have multiple cancer diagnoses. A total of 26,366 people (11.9%) had a diagnosis for at least one additional cancer type beyond the six cancer types specifically included in TORCH-VIC, with these additional diagnoses occurring at various timepoints either before or after their primary TORCH-VIC cancer diagnosis.

**Table 4:** Overview of incident cases, age at diagnosis, derived stage, follow-up time, all-cause deaths, and overall survival for each cancer type

	**Prostate ^a^**	**Breast ^b^**	**Colorectal ^c^**	**Lung ^d^**	**Melanoma ^e^**	**Lymphoid leukaemia ^f^**
**Incident cases, n (%)**	62 120 (100)	52 634 (100)	47 145 (100)	36 207 (100)	31 896 (100)	7 087 (100)
2010	5 130 (8.3)	3 595 (6.8)	3 877 (8.2)	2 487 (6.9)	2 298 (7.2)	388 (5.5)
2011	4 880 (7.9)	3 819 (7.3)	3 868 (8.2)	2 633 (7.3)	2 107 (6.6)	382 (5.4)
2012	4 942 (8.0)	3 820 (7.3)	3 773 (8.0)	2 850 (7.9)	2 316 (7.3)	377 (5.3)
2013	4 437 (7.1)	4 195 (8.0)	3 854 (8.2)	2 836 (7.8)	2 377 (7.5)	419 (5.9)
2014	4 247 (6.8)	4 367 (8.3)	3 838 (8.1)	2 933 (8.1)	2 501 (7.8)	489 (6.9)
2015	4 531 (7.3)	4 449 (8.5)	3 973 (8.4)	2 831 (7.8)	2 737 (8.6)	621 (8.8)
2016	4 963 (8.0)	4 482 (8.5)	4 028 (8.5)	3 131 (8.6)	2 876 (9.0)	721 (10.2)
2017	5 420 (8.7)	4 656 (8.8)	4 108 (8.7)	3 153 (8.7)	3 040 (9.5)	800 (11.3)
2018	5 399 (8.7)	4 878 (9.3)	4 187 (8.9)	3 290 (9.1)	3 180 (10.0)	826 (11.7)
2019	6 249 (10.1)	4 827 (9.2)	4 025 (8.5)	3 365 (9.3)	2 931 (9.2)	717 (10.1)
2020	5 604 (9.0)	4 485 (8.5)	3 774 (8.0)	3 373 (9.3)	2 658 (8.3)	678 (9.6)
2021	6 318 (10.2)	5 061 (9.6)	3 840 (8.1)	3 325 (9.2)	2 875 (9.0)	669 (9.4)
**Diagnosis age (years)**						
Mean (SD)	69 (10)	61 (14)	69 (14)	71 (11)	64 (16)	69 (14)
Range Q1,Q3	62, 75	51, 71	60, 80	64, 80	54, 76	61, 79
**Derived stage, %**						
Stage I	10.9	43.0	21.7	1.09	71.7	–
Stage II	32.05	35.0	22.7	0.70	16.5	–
Stage III	22.35	9.16	21.4	1.34	4.84	–
Stage IV	4.4	4.05	17.1	13.3	3.27	–
Unknow / missing	30.32	8.76	17.1	83.6	3.66	100
**Follow-up (years)**						
Mean (SD)	5.96 (3.66)	6.08 (3.55)	4.82 (3.70)	2.19 (2.14)	5.98 (3.50)	4.95 (3.32)
Range Q1,Q3	2.96, 8.85	3.14, 8.83	1.72, 7.48	0.27, 3.07	3.08, 8.60	2.29, 7.08
**Overall survival, % (95% CI)^g^**						
1-year	94.4 (94.0–94.9)	94.4 (93.3–94.9)	81.4 (80.7–82.2)	53.5 (52.5–54.5)	92.5 (91.8–93.2)	88.3 (86.8–89.9)
5-year	59.8 (58.6–61.1)	54.0 (52.6–55.5)	32.5 (31.4–33.6)	9.9 (9.2–10.6)	56.3 (54.8–57.9)	50.6 (47.6–53.8)
**Deaths, n (%)**	14 048 (22.6)	10 054 (19.1)	21 564 (45.7)	28 681 (79.2)	7 249 (22.7)	2 465 (34.8)
**Total cancer diagnoses, n (%)^h^**						
1	52 403 (84.4)	47 218 (89.7)	39 182 (83.1)	30 730 (84.9)	26 035 (81.6)	5 616 (79.2)
2	8 729 (14.1)	5 035 (9.6)	7 089 (15.0)	4 839 (13.4)	5 194 (16.3)	1 297 (18.3)
≥ 3	988 (1.6)	381 (0.7)	874 (1.9)	638 (1.7)	667 (2.2)	174 (2.4)

CI: confidence interval; ICD-10: International Classification of Diseases, Tenth Revision; SD: standard deviation.

The first 3 digits of the ICD-10 code of tumour diagnosis was used to identify cancer types: ^a^Prostate C61; ^b^Breast C50; ^c^Colorectal C18, C19, C20; ^d^Lung C33, C34; ^e^Melanoma C43; ^f^Lymphoid leukaemia C91.

^g^Overall survival estimated using the Kaplan-Meier method; 95% CI calculated using the Greenwood formula. Individuals alive at last contact with the Victorian Cancer Registry were censored at that date.

^h^Number of cancer diagnoses: for each cancer type, the total number of any other cancer diagnoses for each person were summed (maximum of 6 diagnoses reported).

The distribution of derived stage at diagnosis (reported by the VCR) is presented in Table 4, with completeness varying across cancer types. Stage data are most complete for breast, colorectal, and prostate cancers, although the proportion with unknown or missing stage varied (8.8% for breast to 30.3% for prostate). The majority of breast cancers were diagnosed at stage I (43.0%) and II (35.0%), while colorectal cancer showed a relatively even distribution across stages, with 17.1% diagnosed at Stage IV. Prostate cancer was most commonly diagnosed at stage II (32.1%) and III (22.4%). Stage data for melanoma are presented from 2018 onwards, showing most diagnoses occur at stage I (71.7%). Stage reporting is limited for lung cancer with incomplete coverage across all years (83.6% unknown) and is missing for lymphoid leukaemia.

Mean follow-up time differed by cancer type, ranging from 2.19 years (SD 2.14) for lung cancer to 6.08 years (SD 3.55) for breast cancer, reflecting both survival differences and diagnosis patterns over the study period. The proportion of individuals who are deceased also varied by cancer type, with lung cancer showing the highest mortality (79.2% deceased), followed by colorectal cancer (45.7%) and lymphoid leukaemia (34.8%). In contrast, breast cancer (19.1%), prostate cancer (22.6%), and melanoma (22.7%) had lower mortality. These figures reflect all-cause mortality during the observation period rather than age-standardised survival rates. Overall survival estimates (Table 4) show the one year survival exceeded 90% for breast, prostate, and melanoma, with breast and prostate cancers showing identical estimates (94.4%). Lung cancer had the lowest survival at both one year (53.5%) and five years (9.9%). At five years, prostate, melanoma, breast, and lymphoid leukaemia showed similar survival (50-60%), while colorectal cancer was lower (32.5%).

**Figure 1:**
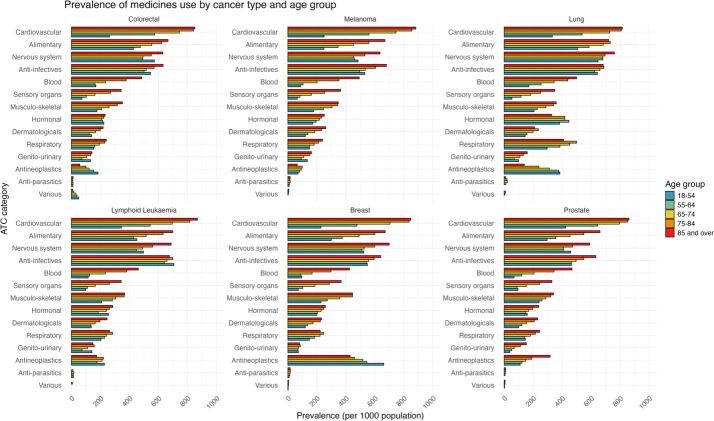
Prevalence of use (≥1 dispensing) of each ATC medicine class, by cancer diagnosis and age group, amongst the TORCH-VIC population in 2022

**Figure 2:**
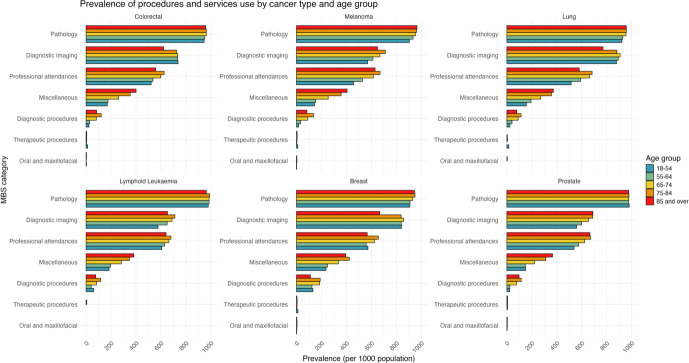
Prevalence of use (≥1 service) of each MBS service and procedure category, by **cancer diagnosis and age group**, amongst the TORCH-VIC population in 2022

Figures 1 and 2 present 2022 prevalence data for the TORCH-VIC cohort. While cohort members were identified through cancer diagnoses recorded between 2010 and 2021, linked administrative data collections extend beyond the cohort identification period (Table 1), enabling observation of ongoing healthcare utilisation.

ATC: Anatomical Therapeutic Chemical classification system. PBS: Pharmaceutical Benefits Scheme. Data represent prevalence of at least one dispensing record in 2022 for each medicine class.

Figure 1 shows the prevalence of prescribed medicine by Anatomical Therapeutic Chemical (ATC) classification [33] medicine class (first level: anatomical main group) for each cancer type and age group in 2022. Overall, cardiovascular system medicines were the most prevalent, dispensed to 69.9% of the cohort, followed by anti-infectives (55.9%) and nervous system medicines (51.7%).

The prevalence of most medicine classes increased with age, except for antineoplastics and immunomodulatory medicines, which decreased with age in most cancer types, with the exception of prostate cancer.

Amongst older people (≥ 65 years), cardiovascular system medicines were the most prevalent, followed by alimentary tract and metabolism medicines, and anti-infectives. In contrast, for younger individuals (< 65 years), the most prescribed class was anti-infectives, followed by nervous system medicines, and antineoplastics and immunomodulatory medicines.

The prevalence of dispensing varied across different cancer types. Lung cancer had the highest dispensing rates for alimentary tract and metabolism, blood and blood-forming organs, systemic hormonal preparations (excluding sex hormones and insulins), and respiratory system medicines. Breast cancer had the highest dispensing rates for antineoplastic and immunomodulatory medicines. Dispensing rates were similar across cancer types for the other medicine classes, including sensory organs, musculoskeletal system, genito-urinary system and sex hormones, dermatologicals, and antiparasitic products.

The prevalence of services and procedures use of each MBS category [34] by cancer type and age group in 2022 is shown in Figure 2. Pathology services had the highest prevalence in the cohort at 95.3%, followed by diagnostic imaging services (72.8%) and professional attendances (60.9%). These patterns were consistent across each cancer type. While the prevalence of most services and procedures was similar across age groups, diagnostic imaging services showed lower prevalence amongst older individuals compared to younger groups, whereas miscellaneous services were more common in the older age groups.

MBS: Medicare Benefits Schedule. Data represent prevalence of at least one service claim in 2022 for each service/procedure category.

## Discussion

Through linkage to the PBS and MBS, TORCH-VIC captures comprehensive data and generates real-world evidence on treatment patterns and therapeutic advancements that have been shown to significantly enhance survival outcomes for many cancers [35]. The longitudinal design of the cohort enables detailed tracking of cancer care trajectories over time, from initial diagnosis through various treatment stages. This approach is essential not only for understanding the direct impacts of treatments but also for assessing associated costs and resource utilisation. Regular updates to TORCH-VIC with new diagnoses and additional follow-up data keep the study relevant and reflective of current practices. TORCH-VIC data can inform the Australian and Victorian cancer plans [36, 37], as well as support healthcare professionals and policymakers to evaluate cancer care outcomes and associated costs in Victoria.

TORCH-VIC has already been used in several projects. These projects include forecasting the number of individuals diagnosed with cancer who are eligible for novel medicines [15, 38], analysing treatment patterns and their associated outcomes, reporting on overall cancer care costs [39], quantifying travel distance and time to radiotherapy treatment centres across Victoria [40, 41], and quantifying immune-related adverse events for Victorians who have received immunotherapy treatment [42].

TORCH-VIC enables diverse research applications. The linked data support evaluation of treatment effectiveness and safety profiles in routine clinical practice, analysis of time to treatment initiation and its relationship with outcomes, and investigation of comorbidity patterns and their impact on treatment decisions and outcomes. The linkage to PBS captures all dispensed medicines across therapeutic classes, enabling research on comorbidity management, polypharmacy, and supportive care alongside cancer treatment, all increasingly recognised as central to cancer outcomes. The population-based design also enables examination of inequities in cancer care access and outcomes by geographic location and socioeconomic status, assessment of adherence to clinical guidelines, optimal care pathways, estimation of the financial burden of cancer care, and modelling the impact of new therapies on population health outcomes.

The increasing incidence of cancer in younger adults represents an emerging public health challenge. TORCH-VIC captures approximately 14,000 individuals diagnosed before age 50 who may face decades of survivorship. The cohort’s linkage infrastructure enables tracking of late effects, fertility impacts, secondary cancers, mental health outcomes, and healthcare utilisation patterns over the life course. This is particularly relevant for understanding treatment-related morbidity that may emerge years or decades post-diagnosis, informing survivorship care models, and quantifying the long-term societal impact of cancer in younger populations. As these individuals age, TORCH-VIC can capture their complete health trajectories, supporting evidence-based survivorship guidelines and health service planning for the growing population of long-term cancer survivors.

TORCH-VIC will integrate additional cancer types and data collections in 2026, to increase its coverage and scope. These include the National Cancer Screening Register, the Australian Immunisation Register, the Australian and New Zealand Intensive Care Society, the Transmission and Response Epidemiology Victoria, the Community Health Minimum Data Set, and the Home and Community Care dataset. The cohort will also expand to include female-specific cancers (vulva, cervix, uterus, ovary), additional solid cancers (brain, liver, bladder, pancreas, kidney, head and neck, oesophagus, stomach), and blood cancers (Hodgkin lymphoma, non-Hodgkin lymphoma, multiple myeloma, and additional leukaemia types).

TORCH-VIC has several limitations. The study focuses on the Victorian population, which could limit the broader applicability of its findings to other regions or across the entire Australian population. While the VAED dataset is robust in capturing hospital services accessed in Victoria, if people seek hospital care outside of Victoria, this will not be reflected in the dataset (inpatient stay). However, all data reported in PBS and MBS are federal and thus captured regardless of the location of care across Australia. Additionally, TORCH-VIC captures only subsidised treatments and services through PBS and MBS. Medicines accessed through clinical trials, compassionate access programs, or private funding are not captured, which may affect the completeness of treatment data particularly for novel therapies. Stage at diagnosis completeness varies across cancer types, with limited reporting for some cancers such as lung, although treatment-based proxies derived from PBS dispensing data and other data collections can be used to infer advanced disease where stage data are incomplete.

Further, current data collections rely on administrative records, which does not include pathology, imaging, test results, or individual-level socio-economic data such as those captured through Census data. The absence of specific lifestyle data and patient-reported outcomes limits the ability to fully understand cancer aetiology, predict treatment outcomes, and assess impacts on quality of life and patient preferences. Finally, observed variations in healthcare utilisation identified through TORCH-VIC may reflect differences in regional care delivery models, patient preferences, and access barriers, which cannot be ascertained using administrative data alone.

TORCH-VIC adds to a growing international landscape of cancer data linkage resources. Australia’s universal PBS and MBS coverage enables capture of all subsidised medicines and medical services across all adult age groups, providing a complementary perspective to these existing data assets. The availability of such resources across diverse healthcare systems creates opportunities for international comparisons of cancer care patterns, treatment outcomes, and health system performance.

## Conclusions

TORCH-VIC provides critical infrastructure for comprehensive real-world evidence on cancer care in Victoria, Australia. Through its comprehensive linkage of population-based cancer diagnoses with prescription medicines, medical services, and healthcare utilisation data, the cohort enables detailed examination of treatment patterns, costs, and outcomes across the cancer care continuum. The methodological framework established through TORCH-VIC can serve as a blueprint for similar initiatives in other Australian states [43, 44], supporting evidence-based policy and practice improvements in cancer care.

## Author contributions

FF, KT, BD, SA, SAP, and MIJ were involved in the development of the original protocol document on which this manuscript was based. SA also provided critical consumer perspective into the data collections for linkage. FF, KT, SAP and MIJ acquired the data. FF prepared and analysed the data. All authors interpreted the data. FF drafted the manuscript. All authors critically reviewed and approved the final manuscript.

## Data Availability

The data are not publicly available due to privacy and ethical restrictions related to health information. TORCH-VIC data is stored within the Secure Unified Research Environment (SURE), which is a remote-access computing environment accessible over encrypted Internet and Australian Academic and Research Network connections, managed by the Sax Institute [45]. Researchers interested in collaborative project proposals can contact the corresponding author for further information. The investigators will review proposals and determine whether specific scientific questions can be addressed and lie within the scope of data custodian and ethical approvals.
